# Transcriptomic signatures of cold adaptation and heat stress in the winter ant (*Prenolepis imparis*)

**DOI:** 10.1371/journal.pone.0239558

**Published:** 2020-10-01

**Authors:** Maria Adelena Tonione, Ke Bi, Neil Durie Tsutsui

**Affiliations:** 1 Department of Environmental Science, Policy, and Management, University of California, Berkeley, Berkeley, California, United States of America; 2 Museum of Vertebrate Zoology, University of California, Berkeley, Berkeley, California, United States of America; 3 Computational Genomics Resource Laboratory (CGRL), California Institute for Quantitative Biosciences (QB3), University of California, Berkeley, Berkeley, California, United States of America; Washington State University, UNITED STATES

## Abstract

Climate change is a serious threat to biodiversity; it is therefore important to understand how animals will react to this stress. Ectotherms, such as ants, are especially sensitive to the climate as the environmental temperature influences myriad aspects of their biology, from optimal foraging time to developmental rate. In this study, we conducted an RNA-seq analysis to identify stress-induced genes in the winter ant (*Prenolepis imparis*). We quantified gene expression during heat and cold stress relative to a control temperature. From each of our conditions, we sequenced the transcriptome of three individuals. Our *de novo* assembly included 13,324 contigs that were annotated against the nr and SwissProt databases. We performed gene ontology and enrichment analyses to gain insight into the physiological processes involved in the stress response. We identified a total of 643 differentially expressed genes across both treatments. Of these, only seven genes were differentially expressed in the cold-stressed ants, which could indicate that the temperature we chose for trials did not induce a strong stress response, perhaps due to the cold adaptations of this species. Conversely, we found a strong response to heat: 426 upregulated genes and 210 downregulated genes. Of these, ten were expressed at a greater than ten-fold change relative to the control. The transcripts we could identify included those encoding for protein folding genes, heat shock proteins, histones, and Ca^2+^ ion transport. One of these transcripts, *hsc70-4L* was found to be under positive selection. We also characterized the functional categories of differentially expressed genes. These candidate genes may be functionally conserved and relevant for related species that will deal with rapid climate change.

## Introduction

Climate change is among the top drivers of biodiversity loss [1] as species or populations will need to either migrate, face extinction, or adapt to the new thermal conditions via phenotypic plasticity or altered behavior [2,3]. Although adaptation to novel environments occurs over longer time spans, climate change has occurred rapidly, thus species may rely heavily on phenotypic plasticity to persist in their environments [4–6]. Concerns over possible effects of climate change has led to interest in temperature-protein interactions. Transcriptomics (RNA-seq) has emerged as a hypothesis-generating tool which can provide insights into the pathways and genes associated with adaptation to thermal conditions [7–9].

Temperature stress causes proteins to misfold, denature, or form aggregates, and this can result in impaired organismal function [10]. In a diverse array of organisms, there is a similar heat shock response (HSR) to combat environmental stress, which typically includes changes in gene expression leading to physiological adjustments [9,11,12]. The HSR involves upregulation of genes that encode for heat shock proteins (Hsps), chaperones or co-chaperones of Hsps, or other genes associated with maintaining proteostasis [e.g. 13–16], and the multigene families of Hsp90, Hsp70, and Hsp40 make up the most prolific proteins in the HSR [17,18]. Some studies suggest that upregulation of genes during physiologically stressful times enhances thermotolerance [19–23]. Alternatively, this response could be a panic or “emergency response,” as higher levels of Hsps have also been associated with reduced thermotolerance [24]. Other studies have noted no upregulation of Hsps with temperature stress [25–27], suggesting an organismal response to heat stress can be more nuanced than simple overall upregulation of Hsps.

Ants occupy diverse thermal environments, across a wide range of latitudes and elevations that encompass many different microclimates and thermal limits. This diversity suggests that adaptation to different temperatures has played an important role in ant evolution [28]. A few previous studies of ants have looked at the role of specific genes in overcoming cold and heat stress. For example, in two of the world’s most thermotolerant ants, *Cataglyphis bombycina* and *C*. *bicolor*, Hsp synthesis begins at lower temperatures and continues during higher temperatures than in the *Formica polyctena*, an ant that inhabits a more moderate environment. This synthesis of Hsps suggests a preadaptation to high temperatures [29]. Many of these focused studies aim to understand expression of stress proteins of Hsp families in response to cold or hot [e.g. 18, 30]. There are very few studies of ants that have attempted to identify genes and pathways associated with a cold or hot stress response without *a priori* information. One recent study looked at gene expression in *Cataglyphis bombycina* during heat stress and documented induction of molecular chaperones [31]. However, we are unaware of studies that have documented genes induced during hot and cold stress in an ant with a preference for cold.

In this study, we use RNA-seq to identify the stress response at the level of gene expression in the winter ant (*Prenolepis imparis*, Say [32]). *Prenolepis imparis* is associated with cooler microhabitats in mesic forests from sea level to high-elevation (2,286 meters) throughout the United States and Mexico, and the southern part of Ontario, Canada [33–36]. *Prenolepis imparis* decreases its activity under warm conditions and is most active in the cooler months (late fall through early spring), when nearly all other ant species exhibit reduced foraging [37,38]. A detailed analysis of their nest structure in Florida found that nest chambers were dug 60 cm below ground, at temperatures between 16 and 24°C [39]. A previous study found populations of *P*. *imparis* have different levels of plasticity and thermal tolerance [40] which makes this species an interesting candidate to examine the genetic basis of these traits.

The overarching goal of this study was to reveal candidate genes necessary for recovery from temperature stress. To do this, we examined the transcriptomic response to short-term temperature stress in *P*. *imparis*. We hypothesized that after three hours at a cold (5°C) or a hot (35°C) temperature, *P*. *imparis* will experience physiological stress and, accordingly, will modulate the expression of genes necessary to survive these stressful conditions.

## Methods

### Sample collection and stress exposure

We collected worker ants from a population on the UC Berkeley campus in Berkeley, California, USA (WGS1984; 122.26317, 37.87281), in June 2014. We chose this site because a previous study showed that *P*. *imparis* from this site exhibit phenotypic plasticity in response to both hot and cold temperatures [40]. After the ants were collected, they were immediately placed in one of three separate thermal conditions: (1) an incubator (Fisher Scientific Isotemp Model 650D Large 600 Series Incubator CAT# 11-690-650D) at 35°C (heat stress), (2) on a room-temperature bench-top approximately 21°C, within the temperature range we expect to find their nests (a control), and (3) a walk-in cold room at approximately 5°C (cold stress). In a preliminary experiment, we placed ten individuals at 36°C, ten individuals at approximately 21°C, and ten individuals at approximately 4°C. After three hours at this temperature, 1–2 individuals in the 36°C and 4°C temperatures were dead. However, when ants were exposed to 35°C and 5°C for three hours, there was no mortality. Therefore, we used the latter temperatures as our experimental conditions. All three groups remained in those conditions for a total of three hours and provided water, but no food, to ensure expression was not influenced by dietary changes. During a two-hour recovery phase, the ants were given a 30% sugar water solution. A two-hour recovery was used because previous studies in *Drosophila* have found that several Hsp genes had a maximum response two hours after the stress [41].

### RNA isolation and mRNA sequencing

After the two-hour recovery phase, ten ants from each temperature condition were collected and ground individually in 1 mL TRIzol (Invitrogen) using a disposable pellet mixer and cordless motor (VWR #47747–370) until homogenized (approx. 15 seconds). RNA extraction was performed according to Rio *et al*. [42], with the following changes: 1) we used 0.1 mL of BCP Phase separation reagent (Molecular Research Center) for every mL of TRIzol and 2) each sample was re-suspended in 28 μL RNAse-free water. Based on Nanodrop concentration estimates, we chose three samples from each treatment that had roughly the same RNA concentration to continue library production; all samples used in library preparation were approximately 35 ng/μL in concentration. The integrity and yield of the RNA extractions were checked by a Bioanalyzer 2100 (Agilent Technologies, Cedar Creek, Texas). All samples had an RNA integrity number (RIN) > 7.0, which indicated quality sufficient for poly(A) selection and cDNA library preparation. Approximately 0.5–2 μg of total RNA was used as the template for cDNA library construction according to manufacturer’s recommendations in the TruSeq RNA Sample Preparation Kit v2 (Illumina: RS-122-2001). The RNA was sheared for eight minutes during the poly(A) selection. To increase the heterogeneity of the cDNA libraries, we split the reaction in half and combined them after enriching the samples for ten cycles. Library quality was assessed using quantitative PCR (qPCR), the Qubit dsDNA High Sensitivity Assay Kit on a Qubit fluorometer, and Bioanalyzer 2100. All nine indexed libraries were normalized to 2 nM then pooled in equal volumes and sequenced using one lane of a 150-bp paired-end Illumina HiSeq2500 run (Vincent J. Coates Genomics Sequencing Laboratory, UC Berkeley).

### *P*. *imparis de novo* transcript assembly and annotation

A total of nine individuals was sequenced and aligned to create a *de novo* transcriptome. Raw reads were filtered using Cutadapt [43] and Trimmomatic [44] to remove low quality reads and adapter sequences. Exact duplicates were eliminated using Super Deduper [45]. After quality control and adapter trimming, reads from all individuals were merged and grouped into clusters based on shared sequence (‘gene’) using Trinity r2014-07-17 [46] on XSEDE [47]. The resulting *de novo* assembly served as a reference with only the longest isoform per gene retained. This reference assembly was annotated against 8 different reference protein databases from other ant species: *Camponotus floridanus*, *Cardiocondyla obscurior*, *Harpegnathos saltator*, *Linepithema humile*, *Pogonomyrmex barbatus*, *Solenopsis invicta*, *Atta cephalotes*, and *Acromyrmex echinatior* [48–53]. The initial round of annotation was done against the NCBI-nonredundant (nr) and SwissProt databases using BLASTX [54] with an e-value threshold of 1e-^10^ and a minimal percent mismatches of 50. The reading frame of each of the matched BLAST hits was then defined by Exonerate [55]. For each reference-specific annotation, when more than one transcript fragment matched against a reference protein, these transcripts were joined together with Ns based on their relative BLAST hit positions to the reference. The resulting annotation from each species was then merged together to purge redundancies. Namely, when the same transcript was annotated with a protein ID from a different reference, only one of the protein IDs was kept. The IDs found here were used in the following gene ontology and functional analyses. All scripts used for cleaning, assembly, and annotation are available on the Computational Genomics Resource Laboratory (CGRL)-QB3 UC Berkeley Github site [56].

### Differential gene expression analysis

We used the quasi-mapping approach implemented in the program Salmon [57]. In this case, the cleaned and trimmed individual reads were quasi-mapped to the merged reference assemblies. To filter out genes that were not expressed or genes with low expression, only genes with a TPM value of ≥ 1 in all the samples were considered. Transcript-level abundance estimates were collapsed to gene-level estimates using *tximport* [58]. Count data were normalized using DESeq2 1.18.1 [59]. The normalized counts were then used by DESeq2 to calculate and plot PCA. We then ran two tests with these normalized data: (1) heat stressed ants (35°C) versus control ants (21°C) and (2) cold stressed ants (5°C) versus control ants (21°C). Transcripts were considered to be differentially expressed (DE) if i) the Benjamini and Hochberg adjusted false discovery rate (FDR) *p*-value was less than 0.01, ii) the absolute value of the fold-change (FC) ≥ 2.0, and iii) the relative standard deviation (RSD) of expression between replicates was less than 0.4 [31].

### Gene ontology and functional annotation

In order to understand the molecular processes relating to temperature-stress, we investigated the functions of genes using the Gene Ontology (GO) database [60,61] and annotated gene metabolic and cellular functions using the Kyoto Encyclopedia of Genes and Genomes (KEGG [62]) pathway maps. To do this, we blasted the DE heat and cold induced transcripts from the DE analysis against the entire NCBI-nr database (e-value < 1e^-5^) and the UniProt [63] annotated protein database. Using the resulting GO annotations, we used WEGO software [64] to functionally classify the terms. The transcripts were also annotated for biochemical pathways [62] using the KEGG Automatic Annotation Server (KAAS) for ortholog assignment and pathway mapping [65] and visualized using iPath3 [66].

### Detection of selection

We tested for selection in the ten most differentially expressed DETs (all upregulated) from the heat stress trials [31] and the four up-regulated DETs detected after the cold stress. Using the coding sequence (CDS) of these DETs, we identified orthologous genes available for other ant species [48–53,67–69] and conducted a selection test using the branch-specific models [70,71] in codeml (packaged in paml version 4.8; [72,73]) as implemented by EasyCodeML [74]. This test is able to detect negative and positive selection by identifying the relative number of non-synonymous and synonymous mutations (ω = *d*_*N*_/*d*_*S*_) relative to expectations under neutral evolution [75]. When at least four ant sequences were available, we were able to conduct a likelihood ratio test using codeml [74]. We compared two *a priori* assumptions: first that a given gene has been evolving at the same rate across all species (the one-ratio model, M0; NSites = 0, model = 0) and second, that a gene is under selection in *P*. *imparis* relative to others (the two-ratio model, BM; NSites = 0, model = 2). We calculated *d*_*N*_/*d*_*S*_ for the *P*. *imparis* branch under the two-ratio model. We included a simplified tree (runmode = 0) from Moreau [76] with only the species for which sequences were included in the analysis. We used twice the difference of the likelihood values calculated under both scenarios to test which model best fit the data and calculated significance using a χ2 distribution (df = 1).

## Results

In this study, we generated nine *P*. *imparis* transcriptome libraries to identify differentially expressed genes during temperature stress. From the nine libraries, the Hi-Seq 2500 run produced a total of 296 million reads of 150bp in length. The total sequence ranged from 29.92 Mb to 37.70 Mb, across the libraries, with means of 32.08 Mb, 33.85 Mb, and 32.75 Mb for the 5°C, 21°C, and 35°C treated ants, respectively. There were no significant differences in the number of sequences between the three datasets (AMOVA, *p* = 0.83). After trimming, we obtained a total of 31.25 Gb of cleaned sequences for further downstream analyses (S1 Table).

### Gene identification and annotation

We performed BLASTX to annotate the transcriptome assembly and inform downstream differential gene expression analysis. After BLASTX annotation, a total of 13,324 contigs had a significant BLAST hit to a gene from one of the previously annotated eight unique ant genomes with an e-value cutoff of 10e^-5^. Altogether, 12,007 contigs (90%) received annotations. Of these, 7,011 contigs (58%) were assigned GO annotations, and 6,122 (51%) were assigned KEGG annotations. Because of the lack of genome information for *P*. *imparis*, only about half of the contigs with hits from the ant genomes were annotated for genes, GO terms, and KEGG numbers (5,991).

### Differential gene expression

**Fig 1:**
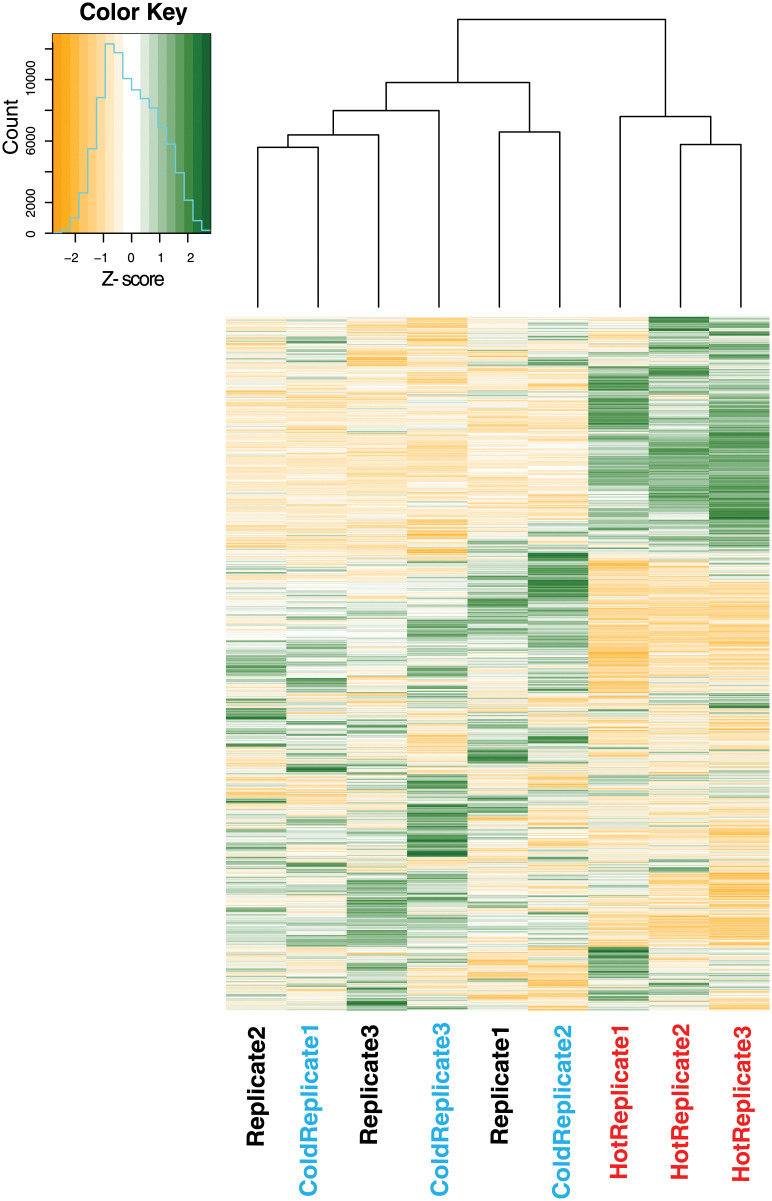
Hierarchical clustering and heatmap of all normalized transcripts. Individual clustering is based on the distance matrix of the similarities between samples. Colors indicate magnitude of change of expression indicated by the z-score calculated from individual transcript levels per gene. Upregulation of a gene is indicated in green, and downregulation is indicated in orange. Individuals are listed at the bottom of the heat map and color coded to indicate their treatment: red = heat-stressed, blue = cold-stressed, black = control.

A total of 8,818 transcripts was tested for differential expression after filtering for low TPM values and collapsing duplicates. Our analysis indicated that 636 transcripts showed DE in response to the heat treatment. Of these, 426 were upregulated, and 210 were downregulated, ten of these transcripts had had a strong increase in expression (≥10 fold-change; FC; Table 1), 426 were upregulated (S2 Table), and 210 were downregulated (S3 Table). In contrast, only seven transcripts differed in expression for cold-stressed ants relative to controls, none of which exceeded 6X FC (Table 2). To summarize our data, we performed hierarchical clustering across all transcripts. The resulting tree clustered the cold-stressed and control ants together, while the heat stressed ants were in their own cluster (Fig 1). PCA analysis on the 8,818 transcripts revealed that most of the variation (60%) was between the heat stressed individuals and the cold stressed and control ants (S1 Fig).

**Table 1:** Proteins that were strongly upregulated after heat stress in *P*. *imparis*.

Best matched gene ID^1^	FC	Protein Product	GO Term^2^
ENSCFLO19236	165.5	Protein lethal(2)essential for life-like	unfolded protein binding (GO:0051082)
ENSCFLO23110	23.0	Heat shock 70 kDa protein cognate 4l isoform X1	ATP binding (GO:0005524)
ENSCFLO19899	18.2	Bag domain-containing protein samui	DNA binding (GO:0003677);metal ion binding (GO:0046872)
ENSSI2.2.0_03007	14.0	Hypothetical protein ALC57_17447	No GO term
ENSSI2.2.0_07291	12.3	Hypothetical protein RF55_12790	No GO term
ENSPB23714	11.01	Calcium-transporting ATPase type 2C member 1 isoform X1	ATP binding (GO:0005524);calcium ion binding (GO:0005509);calcium-transporting ATPase activity (GO:0005388);manganese ion binding (GO:0030145);manganese-transporting ATPase activity (GO:0015410);signal transducer activity (GO:0004871)
ENSCobs_00196	10.86	Aryl hydrocarbon receptor nuclear translocator homolog X4	No GO term
ENSCFLO11886	10.60	Uncharacterized protein LOC105670407 isoform X1	No GO term
ENSLH21966	10.51	Histone H2A	DNA binding (GO:0003677);protein heterodimerization activity (GO:0046982)
ENSCFLO11320	10.13	Hypothetical protein RF55_21065	No GO term

Expression levels are based on transcript counts found at a ≥ 10 fold-change (FC) in individuals after heat stress relative to control.

^1^Contig name based on BLASTx annotation

^2^Based on the biological process

**Table 2:** Proteins that have been differentially expressed after cold stress in *P*. *imparis*.

Best matched gene ID^1^	FC	Direction	Protein product	GO Term^2^
ENSCFLO15808	5.5	Upregulated	Chitinase 3-like	chitin binding (GO:0008061);chitinase activity (GO:0004568)
ENSCFLO19140	3.7	Upregulated	Laccase-4-like isoform X1	copper ion binding (GO:0005507);hydroquinone (GO:0052716);
ENSCFLO11891	3.1	Upregulated	Retrovirus-related Pol polyprotein from transposon 17.6	aspartic-type endopeptidase activity (GO:0004190);endonuclease activity (GO:0004519);nucleic acid binding (GO:0003676);RNA-directed DNA polymerase activity (GO:0003964);
ENSPB20862	3.0	Upregulated	Uncharacterized protein LOC105454034	No GO term
ENSCFLO16203	4.3	Downregulated	Transcription termination factor 2	ATP binding (GO:0005524);DNA binding (GO:0003677);DNA-dependent ATPase activity (GO:0008094);helicase activity (GO:0004386);
ENSCobs_13302	3.3	Downregulated	Putative nuclease HARBI1	No GO term
ENSHSAL27642	2.3	Downregulated	Uncharacterized protein LOC105453329	No GO term

Expression levels are based on transcript counts in individuals after cold stress relative to control.

^1^Contig name based on BLASTx annotation

^2^Based on the biological process

### Gene ontology and functional annotation

**Fig 2:**
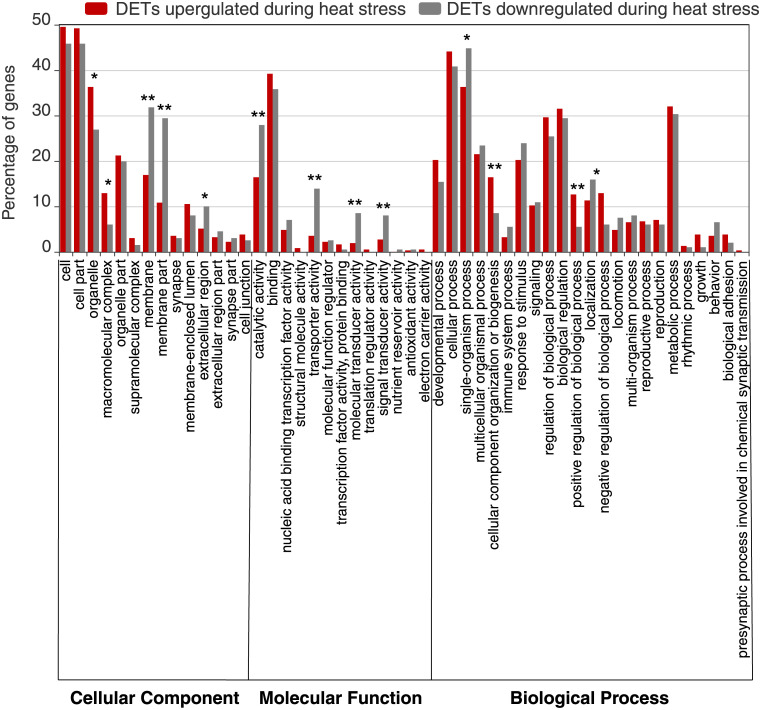
GO functional classification of heat induced DETs. The DETs were placed into three main GO categories: cellular component, molecular function, biological process. Asterisks indicate significant difference (* indicates *p* < 0.05, ** indicates *p* < 0.01).

All transcripts were assigned GO or KEGG numbers based on sequence homology. For the heat induced transcripts, 325 (51%) of the 636 DETs could be assigned GO numbers. GO functional classification of these DETs revealed that 199 (47%) of the 426 upregulated DETs could be categorized into 48 functional groups (Fig 2). Across all the GO domains, we found a high percentage of genes in cell, organelle, cell part, binding, cellular process, biological regulation, and metabolic process. The functional classification of the downregulated DETs resulted in 124 (59%) of the 210 DETs categorized into 46 functional groups. Across all the GO domains, we found a high percentage of genes in cell part, cell, membrane, binding, metabolic process, single-organism, and cellular process. Compared to the downregulated genes, we saw significantly increased expression in the upregulated genes in four functional groups (organelle, macromolecular complex, cellular component organization or biological process, and positive regulation of biological process; Fig 2), while we saw decreased expression in nine functions in the downregulated DETs relative to the upregulated DETs (membrane, membrane part, extracellular region, catalytic activity, transporter activity, molecular transducer activity, signal transducer activity, single-organisms process, and localization; Fig 2). These results indicate that multiple biological processes are necessary for a heat response.

**Fig 3:**
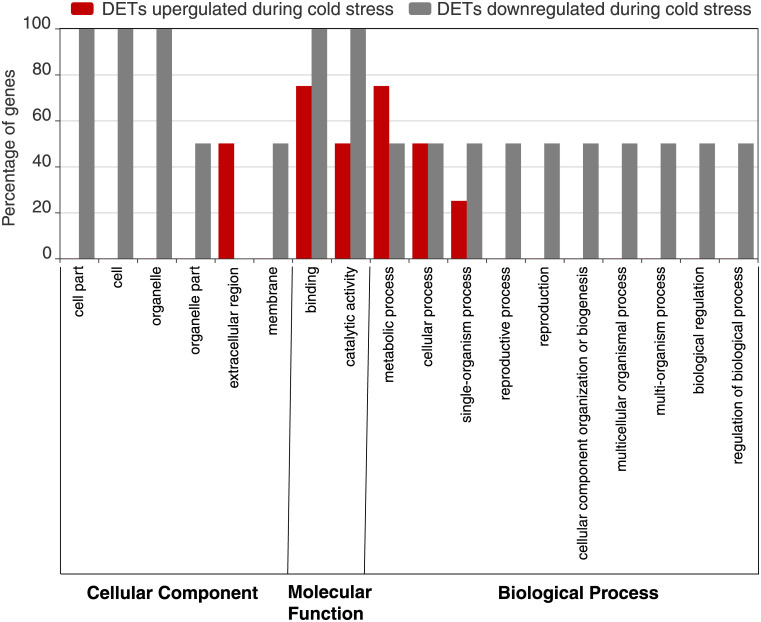
GO functional classification of cold induced DETs. The DETs were placed into three main GO categories: cellular component, molecular function, biological process.

For the cold-induced transcripts, five (71%) of the seven DETs could be assigned GO numbers. GO functional classification of the upregulated DETs revealed that three (75%) of four could be categorized into six functional groups as shown in Fig 3. While GO functional classification of the downregulated DETs revealed that two (67%) of the three DETs could be classified into 17 functional groups (Fig 3). Due to the low number of cold-induced DETs, we did not have enough power to statistically compare the relative numbers of transcripts in each category.

To further evaluate functional pathways associated heat stress or cold stress, we annotated the 643 DETs with KEGG numbers using KASS. KASS identified 132 upregulated and 71 downregulated DETs after heat stress. Using KEGG Pathway Mapper, for the upregulated and downregulated DETs, we found the main pathway involved was metabolic pathways (upregulated; 14, downregulated; 11; S2 Fig). Only one DET was annotated from the cold stress trial: transcription termination factor 2, which mapped to thyroid hormone synthesis (S3 Fig).

### Detection of selection

We were able to test for selection (*d*_*N*_/*d*_*S*_ ratios) in five of the ten most heat induced transcripts and in two of the four cold induced transcripts. For the transcripts that could be tested, a signal of positive selection was detected for the CDS of the heat shock protein, Hsc70-4L (*d*_*N*_/*d*_*S*_ ratio 9.5 times larger leading to the *P*. *imparis* branch; Table 3).

**Table 3:** Likelihood ratio test evaluating signals of positive selection along the *Prenolepis* branch.

Gene	Model	all ants ω	*Prenolepis* ω	Ln L	ratio	LRT P-value
*l(2)efl*	BM	0.094	0.167	-2479.56	0.94	n.s.
	M0	0.097	-	-2480.03		
*Hsc-70-4L*	BM	0.014	0.088	-4459.78	9.48	**
	M0	0.015	-	-4464.52		
*Samui*	BM	0.039	2.383	-411.20	0.00	n.s.
	M0	0.029	-	-411.20		
*Histone 2A*	BM	0.0001	0.0001	-1233.61	0.00	n.s.
	M0	0.0001	-	-1233.61		
LOC105454034	BM	0.310	0.391	-1220.56	0.08	n.s.
	M0	0.315	-	-1220.60		
*Chitinase 3*	BM	0.264	2.383	-1002.36	1.02	n.s.
	M0	0.273	-	-1002.87		
*Laccase-4*	BM	0.181	0.126	-6613.77	1.38	n.s.
	M0	0.176	-	-6614.46		

** indicates p < 0.01, df = 1

## Discussion

In this study, we examined the gene expression and associated pathways used by the winter ant (*Prenolepis imparis*) following heat or cold stress. We found that cold stress produced virtually no changes compared to room temperature controls, indicating that this temperature was not extreme enough to produce a strong physiological response, likely due to the preference of this species for cooler temperatures, which is well-documented [33–35,77]. In contrast to the cold-stress treatment, the heat-stress treatment resulted in altered expression of 636 transcripts, and some of these transcripts displayed enormous changes in the magnitude of expression. The vast majority of the differentially expressed genes found here displayed upregulation rather than downregulation, consistent with heat stress triggering an active genetic response rather than a silencing of baseline gene expression. Many of these genes have been implicated in thermal stress response in other species (see below) and are thus likely involved in the physiological response of *P*. *imparis* to heat.

### Response to heat stress

Because of the stress that heat puts on proteins, it is not surprising that we see the enrichment of gene functional categories for response to temperature and protein folding. All organisms have *Hsp* genes and while the relative amounts of *Hsps* vary between organisms they are among the first lines of defense preventing irreversible denaturation of proteins [13]. In this study, we found the highest levels of expression changes in the gene *lethal(2)essential for life-like (l(2)efl)*, which encodes a small heat shock protein (sHsp). Previous studies have found this gene upregulated in response to both heat [16,31,78] and crowding [14] and downregulated during pathogen stress [79]. In our study, expression levels of this gene were 165-fold higher in the high temperature treated ants relative to the control. In ants, this gene was previously found to be strongly heat inducible in the Sahara-dwelling ant, *Cataglyphis bombycina* [31], but interestingly, not heat inducible in *Aphaenogaster picea* (from more temperate eastern North America) and *Pogonomyrmex barbatus* (from the desert southwest of the United States [18]). Based on the high levels of *l(2)elf* we detected during heat stress, it appears to be one of the most important genes in recovering from this stress.

There was also upregulation in other *Hsps*. We detected *Heat shock 70 kDa protein cognate 4L* (*Hsc70-4L*) 23X higher during heat stress. *Hsc70-4* has been implicated in heat stress response [11,16,24,31,78,80–83] and cold stress response [81,82,84] in many other organisms. Previous studies have shown that ants have divergent copies of *Hsc70-4* that are under positive selection and in *C*. *bombycina*, the Hsp70 protein family was highly induced during heat stress [31]. We also detected a 10X FC increase in *aryl hydrocarbon receptor nuclear translocator* (*ARNT*), a gene which codes for a protein that forms a complex with Hsp90 [85,86], a 7X FC increase in *Hsp70-4L*, a 4X FC increase in *Hsp90*, and a 3X FC increase in *Hsp60*.

Bcl-2-associated athanogene (BAG)-family proteins are a multifunctional group that contribute to a range of physiological processes, including the cell cycle, apoptosis, and stress response [87,88]. In this study, we found substantially elevated levels *Samui*, a gene in the BAG-family, under heat stress. This is not surprising given that the protein Samui is known to interact with Hsp70 to regulate its activity [14,87], and has been shown to increase during heat stress in the damselfly, *Ischnura elegans* [16]. Other studies have found a homologous gene, *Starvin* (*Stv*) associated with both heat stress and cold stress, muscle maintenance, and food uptake [16,89–93]. *Samui* was the only BAG-family member found to be upregulated in our study.

In addition to reduced fitness and impaired function caused by damaged protein structure or protein aggregates, heat stress can also cause oxidative stress at the cellular level, creating transcription errors [94,95]. Histones are crucial in conferring resistance to this DNA damage [96–98]. In our study, we saw greater than a 10-fold increase of expression levels of the gene that encodes for Histone H2A indicating that these proteins are integral to the stress response in *P*. *imparis*. Interestingly, increased expression in *Histone H2A* during heat stress has been found in *Leishmania* parasites as well [99].

In response to heat, we also noted a large expression difference in a transcript relating to calcium ion transport, *Calcium-transporting ATPase type 2C member 1* (*ATPC21*).

Calcium signaling has been suggested as a rapid response to low temperatures: calcium signals begin the temperature-hardening pathways, which induces a number of physiological changes necessary to enhance cold tolerance [100]. Genes relating to maintenance of calcium ion homeostasis have been shown to increase in response to heat stress [26,31]. Truebano *et al*. [101] found that genes involved in Ca^2+^ signaling were increased in the Antarctic bivalve (*Laternula elliptica*) during cold stress, indicating the calcium ions could be biomarkers of the physiological state of the individuals during heat stress as well as cold stress. The high levels of *ATPC21* that we observed in the heat-stressed ants suggest that it might play a key role in initiating the physiological response to heat and inducing a heat hardening pathway.

Finally, among the top upregulated genes, we found four transcripts that were either uncharacterized or hypothetical. Additional work is needed to understand the function of the unknown proteins found here and, in particular, why they are recovered with such large expression differences in the heat stressed individuals.

For the five heat-induced genes that we were able to test for signals of selection, only *Hsc70-4L* harbored signals of positive selection along the *Prenolepis* lineage. A previous study showed that the thermotolerant *Cataglyphis* lineage was also characterized by heat induction of several HSPs and signals of positive selection on *Hsc70-4 h2* and *Hsc70-5* relative to other ant lineages [31].

The biological processes increased during recovery to heat stress (“organelle”, “macromolecular complex”, “cellular component organization or biogenesis” and “positive regulation of biological process”) may indicate the heat-stressed individuals expend energy in cellular repair and protein modification post stress. This is expected given that the heat stress will cause damage to proteins within the cells. In response to the heat, many biological processes were decreased during the recovery time. It is counterintuitive that these processes relate to the cellular membrane, outside the cellular membrane, and cellular transportation; it is expected these cellular components are vulnerable to thermal damage and therefore we would expect increased activity in these areas [102]. However, heat temporarily decreases membrane fluidity, and influence lipid-protein interactions [103] and perhaps they have not had time to adequately recover from the negative consequences of heat stress.

### Response to cold stress

Similar to heat stress, cold stress can also cause denatured or misfolded proteins, leading to harmful aggregates and impaired function. Cold stress may also cause ion imbalance, impairment of cellular metabolism, depletion of cellular ATP, and buildup of toxic metabolic end products [100]. Therefore, we expected transcripts relating to these processes to be over-expressed in cold-stressed individuals. Our results, however, showed few differences between cold-stressed and control individuals, as depicted by the PCA (S1 Fig), heatmap (Fig 1), and DETs (Table 2). This could indicate that our experimental temperature was not low enough to elicit a strong physiological response or that the cold adaptations of *P*. *imparis* are not manifested through the ability to dramatically upregulate genes that help them resist cold. Instead, they may have constitutive mechanisms that allow them to shrug off cold temperature without having to upregulate or downregulate many genes. This is an interesting contrast to the heat-adapted *Cataglyphis*, which showed numerous upregulated and downregulated genes in order to resist the heat [31]. Both the PCA and heatmap show the cold-stressed individuals and control clustering together or within the same group. Additionally, we only found seven DETs with minor expression changes. Transcripts with increased expression that we were able to identify were: *Chitinase 3-like*, *Laccase-4-like isoform X1*, and *Retrovirus-related Pol polyprotein from transposon 17*.*6*.

Chitinase enzymes are involved in the biological function of chitin degradation [104]. They are necessary for a wide range of physiological functions such as immunity, digestion, and phagosytosis [105–107]. Chitinase related genes have been induced during cold stress in beetles [108], and there is evidence of positive selection in chitinase genes in high-altitude Lepidoptera in the genus *Gynaephora* [109]. Together with these studies, the increased expression that we detected in *Chitinase 3-like* indicate that chitinase enzymes are likely important during cold thermal stress.

The peritrophic matrix (PM) is a lining found in the midgut of insects. This semi-permeable layer forms a protective barrier that prevents invasion by pathogens as well as maintaining gut homeostasis and gut integrity [110,111]. Within the midgut PM, Lang *et al*. [112] detected laccases and linked them to oxidation of toxic materials in preparation for excretion. In this study, we detected increased expression in *Laccase-4-like isoform X1* indicating gut osmoregulation as a cold stress response. Interestingly, proteins necessary for gut osmoregulation have been implicated in other stress responses including cold and hot stress, as shown by increased expression in the *mucin* gene in damselfly and *Frost* gene in *Drosophila* [16,91,113], indicating that gut homeostasis could be integral to stress survival.

We found *Retrovirus-related Pol polyprotein from transposon 17*.*6* differentially expressed in this study. This is not surprising given that other studies have detected genes involved in retrotransposon activity and other transposable elements (TEs) upregulated in response to heat [16,114–116]. In addition, retrotransposon genes have been found to be under selection in urban populations of the black ant, *Lasius niger* [117], perhaps assisting in adaptation [114] to the warmer conditions experienced by urban populations [118].

### Conclusions

The molecular mechanisms behind recovery from temperature stress are complex. In this study, we looked at gene expression levels over the entire ant body at one time-point. Other studies have noted that different organs have different expression patterns [119,120], which can even vary over different time-points [27,41] and development stages [120]. Therefore, a productive next step would be to investigate the responses of these genes and others at multiple time points, either by RNA-seq or a quantification method such as qPCR.

Our analysis has revealed a clear pattern of transcriptome change in response to heat stress and identified a number of candidate loci that may be directly involved in resisting thermal stress. A productive next step would be to directly test the functionality of these genes using methods such as RNA interference (RNAi) to suppress the expression and compare the resulting phenotype with the unmanipulated phenotype [22,121]. It is unclear from our study if these expression changes are an adaptive response or the large transcriptomic response is actually a signal of more stress [9,122,123]. Future studies should focus on a functional link between the candidate genes proposed here and thermal tolerance.

Our transcriptomic analysis provides an investigation of the gene expression profiles involved in recovery from heat and cold in *P*. *imparis*. The DETs and pathways identified here could further facilitate investigations into the detailed molecular mechanisms and provide a foundation for future studies of response to temperature stress in *P*. *imparis* or a related species with conserved genes.

## Supporting information

S1 Fig Principal Component Analysis (PCA) of all annotated transcripts used in differential expression analysis for *P*. *imparis*. Expression levels were checked in nine individual ants two hours post a three-hour temperature treatment. Each point represents one individual; transcripts from ants that were subject to the 5°C treatment are shown as blue squares, transcripts from ants that were subject to the 21°C control are shown as white circles, and finally those transcripts from ants that were subject to the 35°C treatment are shown as red triangles.
(EPS)

S2 Fig KEGG pathways with KEGG IDs upregulated during heat stress. The pathways are highlighted in red.
(EPS)

S3 Fig KEGG pathways with KEGG IDs downregulated during heat stress. The pathways are highlighted in red.
(EPS)

S1 Table Reads obtained for each *P*. *imparis* transcriptome sequenced, before and after trimming. (DOCX)

S2 Table Differentially expressed genes after heat stress in *Prenolepis imparis*. (CSV)

S3 Table Differentially expressed genes after cold stress in *Prenolepis imparis*. (CSV)
