# Supplementary material for: Transcriptomic signatures of cold adaptation and heat stress in the winter ant (Prenolepis imparis)
Source: PLoS One. 2020 Oct 1;15(10):e0239558. doi: 10.1371/journal.pone.0239558 (PMC7529264; doi:10.1371/journal.pone.0239558)
Supplement: S1 Table — (DOCX) [file pone.0239558.s004.docx]

Transcriptomic signatures of cold adaptation and heat stress in the winter ant (*Prenolepis imparis*)

Maria Adelena Tonione^1,^*, Ke Bi^2,3^, Neil Durie Tsutsui^1^

^1^ Department of Environmental Science, Policy, and Management, 130 Mulford Hall, #3114, University of California-Berkeley, CA 94720-3114, USA

^2^ Museum of Vertebrate Zoology, University of California, Berkeley, 3101 Valley Life Sciences Building, Berkeley, CA 94720, USA

^3^ Computational Genomics Resource Laboratory (CGRL), California Institute for Quantitative Biosciences (QB3), University of California, Berkeley, Berkeley, CA 94720, USA

^*^Corresponding author

E-mail: [riatoni@berkeley.edu](mailto:riatoni@berkeley.edu) (MAT)

**Supporting Table**

**Table S1. Reads obtained for each *P. imparis* transcriptome sequenced, before and after trimming.**

| **Replicate** | **Sample ID** | **treatment** | **Number of raw reads, million** | **Read length, bp** | **Number of reads after trim, million** | **Average length after trim, bp** | **Total bases after trim, Gb** |
| --- | --- | --- | --- | --- | --- | --- | --- |
| Replicate1 | Toni036 | 21°C | 30.51 | 150 | 24.28 | 136.3 | 3.31 |
| Replicate2 | Toni038 | 21°C | 30.40 | 150 | 24.14 | 118.3 | 2.86 |
| Replicate3 | Toni039 | 21°C | 35.34 | 150 | 27.83 | 132.3 | 3.68 |
| ColdReplicate1 | Toni046 | 5°C | 36.36 | 150 | 29.31 | 143.8 | 4.21 |
| ColdReplicate2 | Toni047 | 5°C | 30.23 | 150 | 24.53 | 125.2 | 3.07 |
| ColdReplicate3 | Toni048 | 5°C | 34.95 | 150 | 28.12 | 142.9 | 4.02 |
| HotReplicate1 | Toni057 | 35°C | 30.61 | 150 | 23.38 | 132.6 | 3.10 |
| HotReplicate2 | Toni060 | 35°C | 37.70 | 150 | 29.90 | 127.0 | 3.80 |
| HotReplicate3 | Toni061 | 35°C | 29.92 | 150 | 24.03 | 133.3 | 3.20 |
